# Molecular Screening via Sanger Sequencing of the Genetic Variants in Non-Alcoholic Fatty Liver Disease Subjects in the Saudi Population: A Hospital-Based Study

**DOI:** 10.3390/metabo12121240

**Published:** 2022-12-09

**Authors:** Faisal Alsaif, Waleed Al-hamoudi, Maram Alotaiby, Amani Alsadoon, Mohammed Almayouf, Hadeel Almadany, Jawahir Abuhaimed, Noman Ghufran, Ahmed Merajuddin, Imran Ali Khan

**Affiliations:** 1 Surgery Department, College of Medicine, King Saud University, Riyadh 12372, Saudi Arabia; 2 Molecular Genetic Pathology Unit, Pathology Department, College of Medicine, King Saud University, Riyadh 12372, Saudi Arabia; 3 Medicine Department, College of Medicine, King Saud University, Riyadh 12372, Saudi Arabia; 4 Laboratories and Blood Bank Services Ministry of Health, Riyadh 12746, Saudi Arabia; 5 Liver Disease Research Center, King Saud University Medical City, Riyadh 12746, Saudi Arabia; 6 Surgery Department, College of Medicine, Prince Sattam bin Abdulaziz University, Riyadh 11942, Saudi Arabia; 7 College of Medicine, Al-Faisal University, Riyadh P.O. Box 400, Saudi Arabia; 8 Research and Development Unit, Adela Inc. 610, University of Avenue, Toronto, ON M5G 2R5, Canada; 9 Department of Clinical Laboratory Sciences, College of Applied Medical Sciences, King Saud University, Riyadh 12372, Saudi Arabia

**Keywords:** NAFLD, NASH, non-NAFLD, PNPLA3, TM6SF2, HFE, Saudi population

## Abstract

Non-alcoholic fatty liver disease (NAFLD) is one of the most common liver diseases, along with steatosis and non-alcoholic steatohepatitis (NASH), and is associated with cirrhosis and hepatocellular carcinoma. Candidate gene and genome-wide association studies have validated the relationships between NAFLD, NASH, *PNPLA3*, *TM6SF2*, and *HFE*. The present study utilized five polymorphisms in three genes: *PNPLA3* (I148M and K434E) *TM6SF2* (E167K), and *HFE* (H63D and C282Y), based on undocumented case–control studies in the Saudi Arabian population. A total of 95 patients with NAFLD and 78 non-NAFLD subjects were recruited. Genomic DNA was isolated, and polymerase chain reaction and Sanger sequencing were performed using specific primers for the I148M, K434E, E167K, H63D, and C282Y. NAFLD subjects were older when compared to controls and showed the significant association (*p* = 0.0001). Non-significant association was found between gender (*p* = 0.26). However, both weight and BMI were found to be associated. Hardy–Weinberg equilibrium analysis confirmed that H63D, I148M, and K434E polymorphisms were associated. Genotype analysis showed only K434E variant was associated with NAFLD and non-NAFLD (OR-2.16; 95% CI: 1.08–4.31; *p* = 0.02). However, other polymorphisms performed with NAFLD and NASH were not associated (*p* > 0.05), and similar analysis was found when ANOVA was performed (*p* > 0.05). In conclusion, we confirmed that K434E polymorphism showed a positive association in the Saudi population.

## 1. Introduction

Non-alcoholic fatty liver disease (NAFLD) is a heterogeneous disorder with multiple metabolic and genetic factors implicated in its pathophysiology, all of which contribute to its progression and development of adverse effects [1]. NAFLD encompasses diseases ranging from simple steatosis to steatohepatitis and is a primary cause of chronic liver damage [2]. In 1986, Schaffner discovered steatosis (triglyceride buildup) within hepatocytes, which progresses to inflammation in non-alcoholic steatohepatitis (NASH). If untreated, it progresses to liver fibrosis, cirrhosis, and possibly hepatocellular carcinoma (HCC) [3,4].

NAFLD is histologically classified as NASH and non-alcoholic fatty liver (NAFL). NASH is defined as the presence of hepatic steatosis and lobular inflammation associated with hepatocyte damage and/or fibrosis [5], whereas NAFL is defined as the presence of 5% hepatic steatosis without evidence of hepatocyte damage. The global prevalence of NAFLD increased from 15% to 25% between 2005 and 2018. The Middle East (32%), South America (31%), and Asia (27%) are the most affected regions worldwide [6]. NAFLD is associated not only with liver damage, morbidity, and mortality, but also with type 2 diabetes mellitus (T2DM), chronic kidney disease (CKD), cardiovascular disease (CVD), and cardiac diseases [7]. It has also been associated with other metabolic disorders, such as obesity, hypertension, and dyslipidemia [8]. NAFLD is a major risk factor for insulin resistance, as evidenced by the increasing prevalence of obesity and T2DM [9]. Obesity is the leading cause of several non-communicable diseases, including NAFLD, and has been identified as a major global threat to public health. However, the vast majority of patients with NAFLD are not obese or even overweight [10,11,12].

Previous studies have highlighted the role of both environmental and genetic factors in the onset and progression of NAFLD [13]. Obesity, T2DM, CVD, and NAFLD are chronic metabolic disorders with a heritable component of vulnerability, accounting for 30–50% of the relative risk. These complex traits are the products of environmental exposures operating on a sensitive polygenic background and are influenced by a number of independent modifiers [14]. Despite the non-genetic factors influencing NAFLD prognosis, evidence suggests a genetic link in the form of genetic risk variants, as evidenced by familial aggregation [15,16,17], twin studies [18], and susceptibility within different ethnicities [19,20,21]. Furthermore, increasing evidence has indicated a significant genetic influence on the formation and development of NAFLD. Genetics holds great potential for risk classification and may lead to future therapeutic intervention [22]. Genome-wide association studies (GWAS) using patatin-like phospholipase domain-containing 3 (PNPLA3), and transmembrane 6 superfamily member 2 genes, both related to NAFLD, have shown unique genetic mediators [23,24]. Additionally, candidate genes, such as hereditary hemochromatosis protein or high iron (*HFE*) gene in NAFLD, which causes hereditary hemochromatosis, have been identified [25].

The prevalence of NAFLD in the general population of Saudi Arabia is 16.6%, 8% of liver donors exhibit steatosis, and 24.9% are obese or have T2DM, leading to rejection of donors. Meanwhile, the prevalence of NAFLD in Saudi Arabian patients with diabetes has been reported to be 55% [26,27]. Many case–control disease-association studies have been conducted to assess the relationship between a variety of genetic variations and NAFLD characteristics. However, conflicting findings on its potential correlation with NAFLD are observed, and the genetic risk factors for NAFLD may differ between populations. To date, no study on five polymorphisms (rs738409, rs2294918, rs58542926, rs1799945, and rs1800562) in *PNPLA3*, *TM6SF2*, and *HFE* associated with NAFLD has been conducted in the Saudi Arabian population. The current study was carried out using Sanger sequencing analysis with *PNPLA3* (rs738409, rs2294918), *TM6SF2* (rs58542926), and *HFE* (rs1799945 and rs1800562) gene polymorphisms in a Saudi population diagnosed with NAFLD.

## 2. Materials and Methods

### 2.1. Ethical Statement

The study protocol was approved by the Institutional Review Board of the College of Medicine at King Saud University (E-17-2654). Additionally, signed informed consent was obtained from 173 Saudi Arabian participants involved in this study. All methods were performed in accordance with the relevant guidelines and regulations (Declaration of Helsinki).

### 2.2. Study Design

In this case–control study, we enrolled 95 patients diagnosed with NAFLD and 78 patients without NAFLD, all subjects recruited from the Division of Gastroenterology, King Saud University (KSU). Adult Saudi Arabian patients with obesity, T2DM, insulin resistance, and ultrasound results demonstrating enlarged/fatty liver were included in this study, while patients diagnosed with viral hepatitis, alcoholic hepatitis, drug-induced hepatitis, alpha-1 antitrypsin deficiency, or Wilson’s disease were excluded. Patients without NAFLD and without any complications were also enrolled in this study, with the inclusion and exclusion criteria for NAFLD subjects.

### 2.3. Anthropometric Measurements

Anthropometric measurements such as age, sex, height, and weight, which were recorded using standardized techniques, were documented. Body mass index (BMI) was calculated as weight in kilograms divided by height in square meters. BMI was categorized as normal (<24.9 kg/m^2^), overweight (25.0–29.9 kg/m^2^), obesity-I (30–34.9 kg/m^2^), obesity-II (35–39.9 kg/m^2^), and obesity-III (>40 kg/m^2^). Additionally, we documented NASH analysis in patients with NAFLD. NASH represents the presence of inflammation and liver damage in addition to fat.

### 2.4. Histological Specimen

A total of 173 liver biopsy specimens were collected based on the histopathological NAFLD assessment score (NAS) from patients with and without NAFLD. The specimens were then fixed in formalin solution, embedded in paraffin blocks, and stained with hematoxylin–eosin and Masson’s trichrome. Patients with NAFLD were classified using the NASH Clinical Research Network Classification based on the liver histology data.

### 2.5. Molecular Screening

Genomic DNA from a liver biopsy specimen was extracted using a Qiagen DNA mini-set, as described previously [28]. The concentration and purity of the extracted DNA were measured using a NanoDrop spectrophotometer. Genotyping was performed using polymerase chain reaction (PCR) with a total of 50 µL reaction containing 24.0 µL of ABI master mix, 5.0 µL of 100 ng the genomic DNA, 1.0 µL of both forward and reverse primers, and 19.0 µL of distilled water. Details for rs738409, rs2294918, rs58542926, rs1799945, and rs1800562 SNPs are found in Table 1. The PCR conditions were as follows: initial denaturation at 95 °C for 5 min, followed by 35 cycles of denaturation for 30 min, annealing at 50–60 °C for 45 s, extension at 72 °C for 45 s, and a final extension at 72 °C for 5 min. The amplified product was electrophoresed using a 2% agarose gel stained with ethidium bromide and visualized via UV transillumination.

### 2.6. Sanger Sequencing Analysis

Sanger sequencing analysis was performed based on, purified amplified products were sequence amplified using the BigDye terminator, and then purified again before bidirectional sequencing using the ABI 3730xl Genetic Analyzer. Analysis was performed using the Sequence Analysis Software version 5.4 and SeqScape version 3.

### 2.7. Statistical Analysis

Continuous variables are presented as mean ± standard deviation, whereas categorical variables are presented as percentages and frequencies. The SPSS software (version 23.0) was used for clinical analysis. The Pearson’s chi-squared test or Fisher’s exact test was used to compare data between the groups. The Pearson’s correlation coefficient was used to calculate the relationships between continuous variables. The chi-squared test was used to compare the Hardy–Weinberg equilibrium (HWE) with one degree of freedom. The Openepi software (version 3.01) was used to calculate genotype and allele frequencies. In addition, Yate’s correction was applied in this study.

## 3. Results

### 3.1. HWE Analysis

The genotype distributions of the five investigated polymorphisms (*HFE*-H63D and C282Y, *PNPLA3*-I148M and K434E, and *TM6SF2*-E167K) were both compatible and incompatible with the controls. H63D (χ^2^ = 0.27; *p* = 0.59), I148M (χ^2^ = 0.05; *p* = 0.81), and K434E (χ^2^ = 1.29; *p* = 0.25) were consistent, whereas C282Y (χ^2^ = 1.00; *p* = 1.00) and E167K (χ^2^ = 7.33; *p* = 0.06) were not consistent.

### 3.2. Clinical Characteristics between NAFLD and Non-NAFLD Subjects

Table 2 shows the anthropometric characteristics of patients with NAFLD and non-NAFLD. The age groups for NAFLD (43.6 ± 11.67) and non-NAFLD (34.9 ± 11.05) were not similar and exhibited a significant association (*p* = 0.0001). In the NAFLD group, 31.7% were males, while 65.3% were females. In the non-NAFLD group, 19.2% were males, while 80.8% were females, which showed a distinct association (*p* = 0.26). However, a non-significant correlation (*p* = 0.46) was observed, with the mean height being practically similar in both cases (153.2 ± 0.08) and controls (151.9 ± 0.09). Both weight and BMI showed significant difference (*p* < 0.05) between the NAFLD (83.9 ± 15.35 and 32.5 ± 6.01, respectively) and non-NAFLD groups (75.7 ± 15.79 and 30.0 ± 5.79, respectively).

### 3.3. Genotyping of the Five SNPs in Patients with and without NAFLD

Table 3 shows the genotypes, genetic modes of inheritance, and allele frequencies of each of the five SNPs. Among the 98 NAFLD cases, the frequencies of the CC, CG, and GG genotypes of SNP I148M were 47.4%, 41.0%, and 11.6%, respectively, whereas in non-NAFLD subjects, the frequencies were 57.7%, 35.9%, and 6.4%, respectively. A positive correlation was not observed in the heterozygous (CG; *p* = 0.309) or variant genotypes (GG; *p* = 0.166). Genetic models such as dominant (OR: 1.515, 95% CI: 0.828–2.770, *p* = 0.176), co-dominant (OR: 1.244, 95% CI: 0.670–2.306, *p* = 0.488), and recessive (OR: 1.912, 95% CI: 0.634–5.759, *p* = 0.243) were carried between cases and controls. The minor allele frequencies of the G allele in the NAFLD and non-NAFLD group were 32.2% and 24.4%, respectively, whereas those of the C allele were 67.8% and 75.6%, respectively. The allele frequency of the I148M variant was not associated with NAFLD incidence (G vs. C, OR: 1.468, 95% CI: 0.912–2.363, *p* = 0.112).

In the NAFLD group, the frequencies of GG, GA, and AA genotypes in K434E were 22.1%, 42.1%, and 35.8%, respectively, whereas in the non-NAFLD group, the frequencies were 23.1%, 56.4%, and 20.5%, respectively. Among the genetic models, only the recessive model (GG + GA vs. AA, OR: 2.160, 95% CI: 1.082–4.312, *p* = 0.027) showed a significant association, while the dominant (GG vs. GA + AA, OR: 1.057, 95% CI: 0.516–2.162, *p* = 0.870) and co-dominant models (GG + AA vs. GA, OR: 0.562, 95% CI: 0.306–1.029, *p* = 0.061) were not associated. The percentages of A and G alleles in the NAFLD group were 56.9% and 43.1%, respectively, whereas in the non-NAFLD groups, the percentages were 48.8% and 51.2%, respectively. A positive association between the allele frequencies and K434E polymorphism was not observed (G vs. A, OR: 1.386, 95% CI: 0.906–2.121, *p* = 0.131).

Genotype and allele frequencies of the E167K polymorphism in *TM6SF2* in the NAFLD and non-NAFLD groups did not show any significant association with any mode of inheritance. The GG genotype frequencies were almost similar in both groups (93.7% vs. 93.6%), while the GA genotypes were varied (6.3% in NAFLD and 5.1% in non-NAFLD). However, in the NAFLD group, the AA genotype was absent, while its frequency was 1.3% in the non-NAFLD group. Dominant (OR: 0.984, 95% CI: 0.288–3.355, *p* = 0.979) and co-dominant models (OR: 1.202, 95% CI: 0.347–4.157, *p* = 0.770) showed similar results; however, in the co-dominant and recessive models, Yates’ correction was applied. The recessive model (OR: 0.270, 95% CI: 0.010–6.733, *p* = 0.393) did not show a statistical association between the cases and controls of NAFLD. The A and G alleles of MAF were 3.2% and 96.8% in the NAFLD group, and 3.9% and 96.1% in the non-NAFLD group, respectively. Finally, allele frequency failed to show a significant association (A vs. G, OR: 0.810, 95% CI: 0.257–2.579, *p* = 0.72).

**Figure 1:**
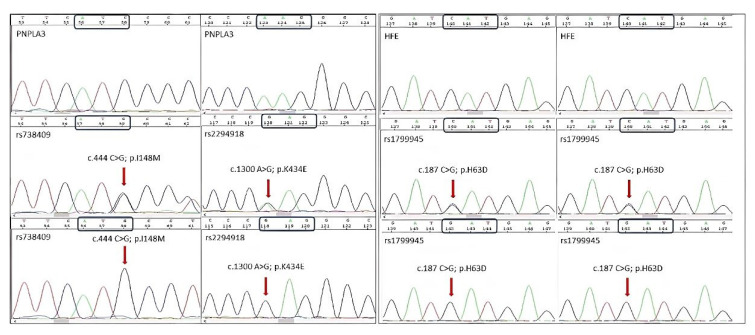
Presentation of Sanger sequencing analysis.

In the NAFLD group, the genotype frequencies of CC, CG, and GG in H63D were 76.8%, 21.1%, and 2.1%, respectively, whereas in non-NAFLD subjects, the frequencies were 69.2%, 26.9%, and 3.9%, respectively. Differences in the proportion of the genetic models were similar with negative associations (CC vs. GG + GC, OR: 0.671, 95% CI: 0.344–1.344, *p* = 0.260; CC + GG vs. CG, OR: 0.723, 95% CI: 0.358–1.461, *p* = 0.366; CC + GC vs. GG, OR: 0.537, 95% CI: 0.087–3.301, *p* = 0.496). The frequency of the G allele in the NAFLD group was 12.7%, which was lower than that in the non-NAFLD group (17.4% (OR: 0.690, 95% CI: 0.380–1.252, *p* = 0.22). All GG genotypes for the C282Y polymorphism in both cases and controls showed 100% frequency. None of the heterozygous or variants showed any genotype for both groups (GA vs. GG or AA vs. GG, OR: 3.697, 95% CI: 0.148–92.01, *p* = 0.393). Allele frequency was also negatively associated (A vs. G, OR: 0.781, 95% CI: 0.015–39.79, *p* = 0.901). Furthermore, a positive or statistical association between the H63D polymorphism in *HFE* and NAFLD was not observed. Yates correction was applied for both E167K (rs58542926) and C282Y (rs1800562) polymorphisms. Figure 1 shows the chromatograms of the SNPs examined in this study.

### 3.4. Clinical Characteristics of Patients with NASH and without NAFLD

Using the Kleiner score, 26.3% (*n* = 25) of patients with NAFLD were classified as having NASH, while 73.7% (*n* = 70) were classified as having NAFLD without NASH. The age and sex distributions of NASH and non-NAFLD showed a significant correlation with age (47.42 ± 10.95 vs. 34.9 ± 11.05; *p* = 0.0003) but not with sex (*p* = 0.24). The height of patients in both groups showed similar results and were not significantly associated (*p* = 0.06). The weight and BMI of patients with NASH and those without NAFLD differed significantly (*p* < 0.05). Table 4 lists the clinical characteristics of both groups.

### 3.5. Genotyping in Patients with NASH and without NAFLD

None of the genotyping analyses of the I148M, K434E, E167K, H63D, and C282Y polymorphisms showed a positive association between the NASH and non-NAFLD groups. Table 5 shows the genotyping, allele frequencies, and genetic modes of inheritance, such as the dominant, co-dominant, and recessive models.

## 4. Discussion

In this hospital-based case–control study, we have shown that the K434E polymorphism is associated with NAFLD and non-NAFLD in the Saudi Arabian population. None of the variants, including K434E, showed positive association with NAFLD or NASH. Additionally, statistical analysis showed a positive association only with obesity and NAFLD (*p* = 0.001).

NAFLD is defined as having fat accumulation in the liver or observing hepatic steatosis via imaging or liver histology when other sources of fat build-up in the liver have been ruled out. Histological examination is essential for the diagnosis of NAFLD [29]. NAFLD and NASH can be confirmed with a liver biopsy. NASH is an advanced stage of NAFLD, a common comorbidity of obesity and T2DM [30]. The prevalence of obesity and T2DM in the Saudi Arabian population is high [31,32], and obesity, T2DM, and NAFLD-NASH are clinically and pathophysiologically connected. Local studies in the Saudi Arabian population have documented various prevalence frequencies of NAFLD. Females are more affected by chronic liver disease than males, which may be due to the expression of sex hormones, which is projected to diminish after menopause [27].

Unfortunately, no medications are allowed for NAFLD treatment; nevertheless, lifestyle changes, including diet and exercise, are effective in managing it [33]. Genes affecting hepatic fat storage, mobilization, and development of NAFLD as variations of transcription factors that control lipid metabolism in the liver and adipose tissue are thus viable candidates for treatment [34]. The major emphasis of investigations has been to identify associations between advanced disease stages and selected SNPs in genes encoding different proteins implicated in disease pathology. Candidate gene association studies are commonly used to examine disease-causing genes in human diseases, and the frequency of candidate genes in one or more known SNPs in patients and controls is evaluated in the quest for a statistical association with NAFLD [35].

Although *PNPLA3* and *TM6SF2* appear to be the most prominent hepatic steatosis determinants across the population, additional genetic deficiencies, which have been relatively infrequent or less significant, have been shown to produce fatty liver metabolism. Genes that control hepatic treatment and VLDL secretion mutations are involved in familial causes of NAFLD [36], Romeo et al. [23] reported that NAFLD is associated with the rs738409 polymorphism in *PNPLA3*. The link between *PNPLA3* and liver histology was validated in patients with NAFLD using GWAS. It was encoded by an isoleucine to methionine substitution variation at protein position 148 (I148M). The I148M polymorphism has been linked to increased hepatic fat accumulation in Europeans regardless of body weight. In a cohort study on the Finnish population, I148M increased the risk of hepatic steatosis [37]. PNPLA3 harbors both triacylglycerol lipase and acylglycerol O-acyltransferase activity, as well as retinyl ester activity in lipid-stellate cells [38]. The interaction between rs2294918 and PNPLA3 mRNA was upregulated, and the protein may be associated with direct effects of PNPLA3 mRNA regulation or PNPLA3 locus methylation on mRNA stability or linkages with other noncoding variants. In 434E allele carriers, PNPLA3 was upregulated [39]. In 2014, Kozlitina et al. [24] validated the relationship between hepatic steatosis and PNPLA3 SNPs and identified polymorphisms in the hepatic triglyceride content gene of TM6SF2. PNPLA3 polymorphisms have been interlinked since 2008 with the risk and severity of NAFLD. Variants of TM6SF2 were also involved in these results [40].

The C-T rs58542926 variant in the *TM6SF2* locus codes for an E to K substitution at position 167, resulting in loss of function, is associated with lower hepatic TM6SF2 mRNA and protein expression. In other tissues, TM6SF2 is mostly expressed in the liver and small intestine [41,42]. Giovanni et al. showed that TM6SF2 rs58542926 can impact the nutrient oxidation, glucose homeostasis, and postprandial lipoprotein of adipokines in patients with NAFLD [43]. Although TM6SF2 does not have a specific function, it affects cholesterol synthesis and lipoprotein secretion [38].

In 1996, Feder et al. initially identified *HFE* on the petite arm of chromosome 6 at 6p21.3, encompassing a 343-amino acid long glycoprotein [44]. Excess iron absorption in the liver hastens the progression of NAFLD to NASH owing to oxidative stress. Iron and heme catalyze oxidation processes caused by reactive oxygen species emitted during Fenton reactions, contributing to oxidative stress [45]. *HFE* has many genetic variants, including two missense mutations: an amino acid replacement from cysteine to tyrosine (C282Y) and a histidine to aspartate substitution (H63D) [46].

Previous studies have reported an association of I148M and K434E polymorphisms in *PNPLA3* with NAFLD in the global population [47,48,49,50,51,52,53]. However, our study was not associated with the I148M polymorphism, but was associated with the K434E variant in *PNPLA3*. Our study is in agreement with previous studies [39,54]. Additionally, limited studies have been performed on the meta-analysis of I148M and K434E polymorphisms in *PNPLA3* [55,56,57]. Furthermore, in our study, the E167K polymorphism was not associated with NAFLD or NASH. However, previous studies have reported positive and negative associations between NAFLD and NASH [24,47,58,59,60,61]. A meta-analysis study on the E167K variant in preventing CAD and conferring risk for NAFLD revealed that the rs58542926 polymorphism is a key regulator of blood lipid characteristics in global studies [62]. Meta-analysis studies have also shown the E167K (rs58542926) polymorphism in *TM6SF2* in NAFLD and other human diseases, such as carcinoma and liver fibrosis [42,62,63,64]. For the H63D polymorphism, 21% of heterozygotes and 2.1% of homozygous variants were present in NAFLD cases in the present study. None of the genotypes was heterozygous or homozygous for variants of the C282Y polymorphism, and no statistical association between the H63D and C282Y polymorphisms in NAFLD was observed. Previous studies showed both associations in NAFLD subjects [65,66,67]. Our study was not in agreement with the documented studies with positive association may be due to the lack of high sample size, or ethnicity playing a major role. The major limitation of our study is the small sample size. We recruited only 95 patients with NAFLD and 78 patients without NAFLD. Nevertheless, recruiting native Saudi Arabian patients and direct sequencing were the strengths of our study.

## 5. Conclusions

In conclusion, we confirmed that K434E polymorphism showed a positive association in the Saudi Arabian population. Further study on the multiple genetic variants associated with NAFLD using a larger sample size is recommended.

## Figures and Tables

**Table 1:** SNPs in the NAFLD and non-NAFLD groups involved in this study.

S. No	Gene	SNP	Rsnumber	Mutation	Amino Acid Substitution
1	HFE	H63D	rs1799945	C-G	Histidine-63-Aspartic acid
2	HFE	C282Y	rs1800562	G-A	Cysteine-282-Tyrosine
3	PNPLA3	I148M	rs738409	C-G	Isoleucine-148-Methionine
4	PNPLA3	K434E	rs2294918	G-A	Lysine-434-Glutamic acid
5	TM6SF2	E167K	rs58542926	G-A	Glutamic acid-167-Lysine

**Table 2:** Anthropometric measurements of NAFLD and non-NAFLD subjects involved in this study.

Anthropometric	NAFLD (*n* = 95)	Non-NAFLD (*n* = 78)	*p* Value
Age (Years)	43.6 ± 11.67	34.9 ± 11.05	0.0001
Gender (Male; Female)	31 (31.7%): 64 (65.3%)	15 (19.2%): 63 (80.8%)	0.26
Weight (Kgs)	83.9 ± 15.35	75.7 ± 15.79	0.0007
Height (Cms)	153.2 ± 0.08	151.9 ± 0.09	0.46
BMI (kg/m^2^)	32.5 ± 6.01	30.0 ± 5.79	0.006

**Table 3:** Genotype and allele distribution in the NAFLD and non-NAFLD groups.

Genes	Rsnumber	Genotype/Alleles	NAFLD (%)	Non-NAFLD (%)	OR	95% CIs	*p* Value
PNPLA3 (I148M)	rs738409	CC	45 (47.4%)	45 (57.7%)	Reference	Reference	Reference
		CG	39 (41.0%)	28 (35.9%)	1.393	0.733–2.649	0.309
		GG	11 (11.6%)	05 (6.4%)	2.200	0.707–6.844	0.166
		CG + GG vs. CC	45 (47.4%)	45 (57.7%)	1.515	0.828–2.770	0.176
		CG vs. CC + GG	56 (59.0%)	51 (64.1%)	1.244	0.670–2.306	0.488
		CC + CG vs. GG	84 (88.4%)	73 (93.6%)	1.912	0.634–5.759	0.243
		C	129 (67.8%)	118 (75.6%)	Reference	Reference	Reference
		G	61 (32.2%)	38 (24.4%)	1.468	0.912–2.363	0.112
PNPLA3(K434E)	rs2294918	GG	21 (22.1%)	18 (23.1%)	Reference	Reference	Reference
		GA	40 (42.1%)	44 (56.4%)	0.779	0.363–1.668	0.520
		AA	34 (35.8%)	16 (20.5%)	1.821	0.766–4.329	0.179
		AA vs. GA + AA	21 (22.1%)	18 (23.1%)	1.057	0.516–2.162	0.870
		GA vs. GG + AA	55 (57.9%)	34 (43.6%)	0.562	0.306–1.029	0.061
		GG + GA vs. AA	61 (64.2%)	62 (79.5%)	2.160	1.082–4.312	0.027
		G	82 (43.1%)	80 (51.2%)	Reference	Reference	Reference
		A	108 (56.9%)	76 (48.8%)	1.386	0.906–2.121	0.131
TM6SF2 (E167K)	rs58542926	GG	89 (93.7%)	73 (93.6%)	Reference	Reference	Reference
		GA	06 (6.3%)	04 (5.1%)	1.230	0.334–4.525	0.754
		AA	00 (0%)	01 (1.3%)	0.273	0.010–6.819	0.398 *
		GA + AA vs. GG	89 (93.7%)	73 (93.6%)	0.984	0.288–3.355	0.979
		GA vs. GG + AA	89 (93.7%)	74 (94.9%)	1.202	0.347–4.157	0.770 *
		GG + GA vs. AA	95 (100%)	77 (98.7%)	0.270	0.010–6.733	0.393 *
		G	184 (96.8%)	150 (96.1%)	Reference	Reference	Reference
		A	06 (3.2%)	06 (3.9%)	0.81	0.257–2.579	0.72
HFE (H63D)	rs1799945	CC	73 (76.8%)	54 (69.2%)	Reference	Reference	Reference
		CG	20 (21.1%)	21 (26.9%)	0.704	0.347–1.427	0.329
		GG	02 (2.10%)	03 (3.90%)	0.493	0.079–3.054	0.438
		CG + GG vs. CC	73 (76.8%)	54 (69.2%)	0.671	0.344–1.334	0.260
		CG vs. CC + GG	75 (78.9%)	57 (73.1%)	0.723	0.358–1.461	0.366
		CC + CG vs. GG	93 (97.9%)	75 (96.1%)	0.537	0.087–3.301	0.496
		C	166 (87.3%)	129 (82.6%)	Reference	Reference	Reference
		G	24 (12.7%)	27 (17.4%)	0.690	0.380–1.254	0.222
HFE (C282Y)	rs1800562	GG	95 (100%)	78 (100%)	Reference	Reference	Reference
		GA	00 (0%)	00 (0%)	3.697	0.148–92.01	0.393 *
		AA	00 (0%)	00 (0%)	3.697	0.148–92.01	0.393 *
		G	190 (100%)	156 (100%)	Reference	Reference	Reference
		A	00 (0%)	00 (0%)	0.781	0.015–39.79	0.901 *

* indicates Yate’s correction.

**Table 4:** Anthropometric measurements of the NASH and non-NAFLD subjects involved in this study.

Anthropometric	NASH (*n* = 25)	Non-NAFLD (*n* = 78)	*p* Value
Age (Years)	47.42 ± 10.95	34.9 ± 11.05	0.0003
Gender (Male; Female)	19 (76%): 06 (24%)	15 (19.2%): 63 (80.8%)	0.24
Weight (Kgs)	85.57 ± 17.23	75.7 ± 15.79	0.009
Height (Cms)	152.8 ± 0.08	151.9 ± 0.09	0.06
BMI (kg/m^2^)	33.48 ± 6.12	30.0 ± 5.79	0.01

**Table 5:** Genotype frequencies with various modes of inheritance in the NASH and non-NAFLD groups.

Genes	Rsnumber	Genotype/Alleles	NASH (%)	Non-NAFLD (%)	OR	95% CIs	*p* Value
PNPLA3 (I148M)	rs738409	CC	12 (48%)	45 (57.7%)	Reference	Reference	Reference
		CG	10 (40%)	28 (35.9%)	0.730	0.301–1.768	0.485
		GG	03 (12%)	05 (6.4%)	1.227	0.268–5.608	0.791
		CG + GG vs. CC	12 (48%)	45 (57.7%)	1.477	0.598–3.648	0.396
		CG vs. CC + GG	15 (60%)	51 (64.1%)	1.190	0.472–3.000	0.711
		CC + CG vs. GG	22 (88%)	73 (93.6%)	1.991	0.440–8.999	0.363
		C	34 (68%)	118 (75.6%)	Reference	Reference	Reference
		G	16 (32%)	38 (24.4%)	1.461	0.727–2.936	0.286
PNPLA3(K434E)	rs2294918	GG	09 (36%)	18 (23.1%)	Reference	Reference	Reference
		GA	13 (52%)	44 (56.4%)	0.590	0.214–1.625	0.307
		AA	03 (12%)	16 (20.5%)	0.375	0.086–1.631	0.182
		GA + AA vs. GG	09 (36%)	18 (23.1%)	0.533	0.201–1.409	0.201
		GA vs. GG + AA	12 (48%)	34 (43.6%)	0.837	0.339–2.066	0.699
		GG + GA vs. AA	22 (88%)	62 (79.5%)	0.528	0.140–1.989	0.339
		G	31 (62%)	80 (51.2%)	Reference	Reference	Reference
		A	19 (38%)	76 (48.8%)	0.645	0.336–1.238	0.186
TM6SF2 (E167K)	rs58542926	GG	24 (96%)	73 (93.6%)	Reference	Reference	Reference
		GA	01 (04%)	04 (5.1%)	0.760	0.081–7.137	0.810
		AA	00 (00%)	01 (1.3%)	1.000	0.039–25.35	0.999 *
		GA + AA vs. GG	24 (96%)	73 (93.6%)	0.608	0.067–5.468	0.654
		GA vs. GG + AA	24 (96%)	74 (94.9%)	0.770	0.082–7.234	0.819
		GG + GA vs. AA	25 (100%)	77 (98.7%)	1.013	0.040–25.65	0.993 *
		G	49 (98%)	150 (96.1%)	Reference	Reference	Reference
		A	01 (02%)	06 (3.9%)	0.510	0.059–4.342	0.530
HFE (H63D)	rs1799945	CC	24 (96%)	54 (69.2%)	Reference	Reference	Reference
		CG	00 (00%)	21 (26.9%)	0.051	0.003–0.889	0.005 *
		GG	01 (04%)	03 (3.90%)	0.750	0.074–7.583	0.806
		CG + GG vs. CC	24 (96%)	54 (69.2%)	0.093	0.011–0.733	0.006
		CG vs. CC + GG	25 (100%)	57 (73.1%)	0.052	0.003–0.899	0.005 *
		CC + CG vs. GG	24 (06%)	75 (96.1%)	1.042	0.103–10.49	0.972
		C	48 (96%)	129 (82.6%)	Reference	Reference	Reference
		G	02 (04%)	27 (17.4%)	0.199	0.045–0.869	0.01
HFE (C282Y)	rs1800562	GG	25 (100%)	78 (100%)	Reference	Reference	Reference
		GA	00 (0%)	00 (0%)	3.078	0.059–159.1	0.556 *
		AA	00 (0%)	00 (0%)	3.078	0.059–159.1	0.556 *
		G	50 (100%)	156 (100%)	Reference	Reference	Reference
		A	00 (0%)	00 (0%)	3.099	0.060–158.2	0.552 *

* indicates Yate’s correction.

## Data Availability

The data presented in this study are available on request from the corresponding author. The data are not publicly available due to privacy.
